# CLIB: Contrastive learning of ignoring background for underwater fish image classification

**DOI:** 10.3389/fnbot.2024.1423848

**Published:** 2024-07-31

**Authors:** Qiankun Yan, Xiujuan Du, Chong Li, Xiaojing Tian

**Affiliations:** ^1^College of Computer, Qinghai Normal University, Xining, China; ^2^Qinghai Provincial Key Laboratory of IoT, Xining, China; ^3^The State Key Laboratory of Tibetan Intelligent Information Processing and Application, Xining, China

**Keywords:** underwater fish image classification, contrastive learning, deep learning, self-supervised visual representation learning, background noise

## Abstract

Aiming at the problem that the existing methods are insufficient in dealing with the background noise anti-interference of underwater fish images, a contrastive learning method of ignoring background called CLIB for underwater fish image classification is proposed to improve the accuracy and robustness of underwater fish image classification. First, CLIB effectively separates the subject from the background in the image through the extraction module and applies it to contrastive learning by composing three complementary views with the original image. To further improve the adaptive ability of CLIB in complex underwater images, we propose a multi-view-based contrastive loss function, whose core idea is to enhance the similarity between the original image and the subject and maximize the difference between the subject and the background, making CLIB focus more on learning the core features of the subject during the training process, and effectively ignoring the interference of background noise. Experiments on the Fish4Knowledge, Fish-gres, WildFish-30, and QUTFish-89 public datasets show that our method performs well, with improvements of 1.43–6.75%, 8.16–8.95%, 13.1–14.82%, and 3.92–6.19%, respectively, compared with the baseline model, further validating the effectiveness of CLIB.

## 1 Introduction

The ocean is one of the most important ecosystems on Earth and is an essential field for human survival and development. However, in recent years, the marine ecosystem has been continuously damaged (Georgian et al., 2022; Jiao et al., 2023). To protect the oceans, we need to understand the health of the oceans, and information such as the distribution of different species of fish and the number of fish in a particular watershed can well reflect the health of the ecological environment in that watershed (Trindade-Santos et al., 2022; Xuan et al., 2022; Yu et al., 2022). Therefore, the study of the species and number of fish through the collected images of underwater fishes is of great significance for further understanding the health of the oceans and protecting endangered species (Ovalle et al., 2022; Zhang D. et al., 2022).

**Figure 1:**
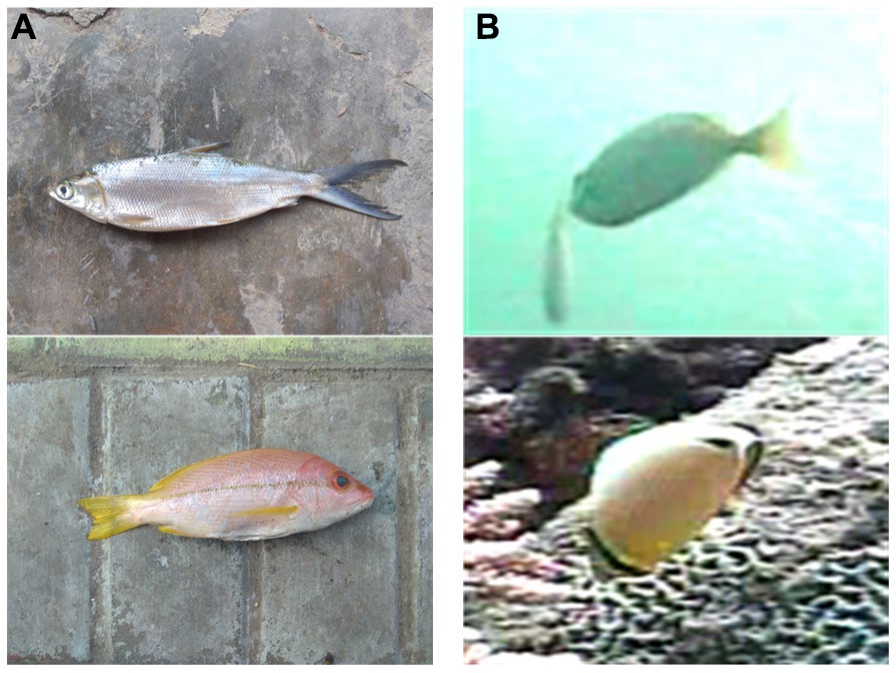
Images taken on land vs. images taken underwater. **(A)** Images taken on land. **(B)** Images taken underwater.

However, underwater optical images are significantly different from land optical images. The lights with different wavelengths have different propagation characteristics in water, resulting in the collected underwater images being characterized by color distortion, visual blurring, and low contrast (Chen et al., 2022; Wang et al., 2022; Lu et al., 2023), which is shown in Figure 1. Figure 1A shows the fish images taken in the terrestrial environment, and it can be seen that the texture of the images is clear and visible, Figure 1B shows the fish images taken in water environment, which are characterized by color distortion, visual blurring, etc. In addition, due to the high mobility of fish, the same species of fish may appear in different backgrounds while different species of fish may appear in the same background, which leads to the background not only having no positive effect but also interfering with the training of the model. The background noise brings about great challenges to the recognition of underwater fish images.

Traditional machine learning methods (Larsen et al., 2009) for underwater fish image classification usually use manually designed features to extract features, and it could be more scalable and generalizable in the face of large-scale datasets. The later emergence of supervised visual representation learning (Li et al., 2021) solved the drawbacks of manually designed features in traditional machine learning methods and attracted much response. However, supervised visual representation learning relies on manually labelled labels when training the model. When facing large-scale underwater fish image datasets, the labelling work on the labels consumes a lot of time and energy for oceanography experts. The emergence of self-supervised visual representation learning (Ericsson et al., 2022) in recent years has improved this problem by requiring only a small number of labels to fine-tune the model to achieve impressive results, significantly reducing the tediousness of labelling data. However, the current self-supervised visual representation learning methods are primarily designed for general-purpose models, which could not work well when facing underwater images of noisy noise. To address the aforementioned issues, this paper proposes contrastive learning of ignoring background for underwater fish image classification called CLIB, which is proposed to reduce the negative impact of background noise. The main contributions of this paper are as follows:

1. This paper reconstructs the view of contrastive learning based on the characteristics of underwater fish images. The subject and background of the image are extracted through an extraction module, and the original image, extracted subject, and background constitute three views for contrastive learning.
2. This paper proposes a multi-view-based contrastive loss function and defines a sample in the subject view as a positive sample of the corresponding image in the original view, while all other samples in the three views are negative samples of the original image.
3. This paper conducts a large number of comparative experiments from three perspectives: different resolutions, complex backgrounds, and few-sample to verify the superiority of the proposed CLIB method in underwater fish image classification.

The rest of the paper is organized as follows. Section 2 reviews existing recognition methods of underwater fish and visual representation methods of self-supervising. Section 3 describes our proposed CLIB method. Section 4 presents the experimental results by comparing the CLIB with the mainstream methods. Section 5 concludes the paper.

## 2 Related work

### 2.1 Traditional machine learning methods

Scholars’ research on fish image classification can be traced back to 1990 (Xu et al., 2020), and most of the early research combined traditional machine learning models with image processing techniques, which mainly focused on the design of feature extraction and improving the accuracy of classification by extracting more favorable information such as shape and texture. For example, Spampinato et al. achieved fish image classification by combining texture features and shape features (Spampinato et al., 2010). Texture features were extracted according to the statistical moments of the grayscale histogram, spatial Gabor filtering, and the properties of the co-occurrence matrix. Shape features were extracted by using curvature scale-spatial transformation and the histogram of boundary Fourier descriptors. Huang et al. achieved fish image classification by extracting 66 features from different parts of a fish composed of color, shape, and texture and reduced the feature dimensions by a forward sequential feature selection procedure (Huang et al., 2012). Fouad et al. described the local features extracted from a set of fish images to differentiate fish species through the algorithm supporting vector machine combined with an accelerated robust feature algorithm based on scale-invariant feature transformation (Fouad et al., 2013). Hu et al. extracted six sets of feature vectors, including the color features of the image, the color features of texture sub-images, the features of statistical texture, and the features of texture based on wavelets, and the feature vectors were fed into the supporting Vector Machine for classification (Hu et al., 2012). Khotimah et al. extracted eight texture and shape features from fish images using image processing methods and then used these features to create a classification model using a decision tree (Khotimah et al., 2015). Most early research on the classification of underwater fish image was based on traditional machine-learning models and image-processing techniques (Zhang et al., 2021; Zhang Z. et al., 2022). The main steps include (1) denoising and enhancing the underwater fish images using image processing techniques, (2) extracting the pre-designed features artificially from the underwater fish images, and (3) training the traditional machine learning models based on the extracted features to classify the fish.

However, the classification effectiveness of traditional machine learning models is closely related to feature extraction methods designed manually, and most researchers rely on experience to design the features, which has the disadvantage of a certain degree of subjectivity and blindness. Although these methods achieve some classification results, most of the features designed by the researchers are designed for only a specific dataset. When faced with a new dataset or applied in practice, the classification results of the model usually have significant errors compared to the reality.

### 2.2 Supervised visual representation learning

With the development of deep learning, deep learning-based methods have achieved good results in various fields in recent years. Different from traditional machine learning methods that rely on hand-designed feature extraction, deep learning methods are capable of automatic feature learning, which dramatically reduces the tediousness of design while improving performance. For example, Sun et al. solved the problem of limited discriminative information in low-resolution images by using deep learning and super-resolution methods to explicitly learn discriminative features in relatively low-resolution images (Sun et al., 2016). Deep et al. used convolutional neural networks to extract features and then used support vector machines and K-Nearest Neighbor to classify images (Deep and Dash, 2019). Based on the idea of contrastive learning, Zhang et al. encouraged the model to learn more discriminative features for different categories of images and similar features for images of the same category (Zhang et al., 2021). In addition, a regularization technique known as attentional suppression was used to prevent the model from paying much attention to the background. To reduce the effect of extreme noise in underwater images, Zhang et al. trained the model using adversarial perturbation images with the perturbation method, which helps to train a better recognition model from images containing extreme noise (Zhang D. et al., 2022). Li et al. used a method of multi-color space coding to fully integrate the feature advantages of different color spaces and then obtained the global and local deep features of the images in multiple dimensions through the multi-channel attention path aggregation strategy, and finally form a multi-channel attention network architecture through the embedding and stacking of multi-channel attention modules, which strengthens the perception of image features (Li et al., 2023).

Although supervised visual representation learning can extract data features, labels pre-labeled manually are still required in feature learning. Labeling data requires a lot of effort and time from the experts, and there may be some labeling errors, which can bring about great misleading in subsequent model learning. Self-supervised visual representation learning (Chen et al., 2020; Sang et al., 2022) solves this problem. With self-supervised representation learning, it is possible to learn the model without knowing the label information of the image. When ported to the downstream task, only a small amount of label information is needed to fine-tune the model to approximate or even exceed the effect of supervised learning.

### 2.3 Self-supervised visual representation learning

Self-supervised visual representation learning can provide powerful deep feature learning without the need for large amounts of labeled data and alleviate the annotation bottleneck to some extent (Ericsson et al., 2022). The most classical self-supervised visual representation learning is contrastive learning, which learns data representation by maximizing the similarity between positively correlated samples and minimizing the similarity between uncorrelated samples. With contrastive learning, a label is first derived from the unlabeled data by a pre-defined strategy, and then the model is trained using this label and the data. The key in contrastive learning is how to design the strategy for deriving a label, which is called a pretext task by scholars. The effectiveness of the pretext task determines the effectiveness of the model for downstream tasks. Consequently, the choice of the pretext task is vital for self-supervised visual representation learning. For example, He et al. thought that increasing the number of negative samples can increase the difficulty of comparison learning and enable the model to learn more detailed feature information (He et al., 2020). Therefore, they proposed the Momentum Contrast (MoCo) learning method, which achieved good results by adding a MEMORY BANK and updating the encoder parameters using momentum. Chen et al. explored the optimal combined method of data augmentation by eliminating memory banks and encouraging larger Batch sizes and longer training times (Chen et al., 2020). Chen et al. presented a self-supervised learning method without negative samples as well as without increasing the batch size (Chen and He, 2021). In the self-supervised learning method, the feature vectors obtained from one of the two-branch networks after passing through the encoder and the feature vectors from another one of the two-branch networks passing through the encoder and the multilayer perceptron are mutual positive samples. The network is trained by maximizing the similarity of the positive sample pairs, which achieved good results.

In addition to the above papers, some excellent methods of self-supervised visual representation learning are available. However, due to the particular characteristics of underwater images, applying general methods to underwater fish image classification does not achieve ideal results. In this paper, from the idea of focusing on the subject and ignoring the background, we innovatively design a contrastive learning of ignoring the background for underwater fish image classification, which is more suitable for underwater fish image classification.

## 3 The CLIB method

**Figure 2:**
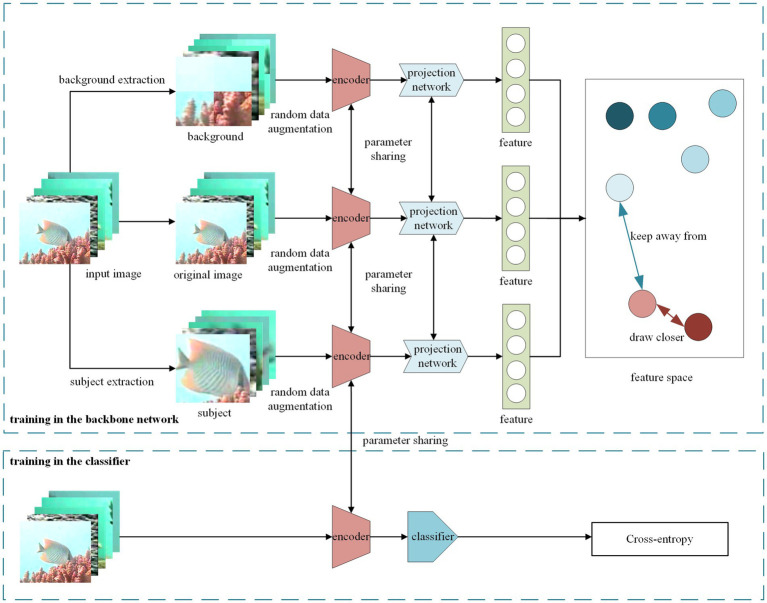
Overview of CLIB training. The upper half of the figure shows the self-supervised training of the backbone network with the CLIB method, and the lower half of the figure shows the classifier training in fine-tuning stage in which the parameters of the trained backbone network are freezed and a classifier is added for supervised training the whole network with a small amount of data.

The training overview diagram of CLIB is shown in Figure 2. The subjects and backgrounds of the input images are first extracted by the extraction module. Then, the original images and the extracted subjects and backgrounds constitute three views, respectively, which are fed into the respective encoders after random data augmentation and then fed into the feature space through the projection head to compute the multi-view-based contrastive loss.

### 3.1 Symbol definition

This paper defines the main symbols as shown in Table 1.

**Table 1:** Main symbols.

Symbol	Meaning
$x\in X\in R^{\left(W*H*C\right)}$	Underwater fish image
W,H	Width and height of image
t∈T	Data augmentation
$f\left(\cdot \right)$	Backbone
$g\left(\cdot \right)$	Projection network
E	Subject and background extraction module

### 3.2 Subject and background extraction module

Before the original image is input into the model, the image is processed to the specified size, and then the subject and background of the image are extracted according to the extraction rules.

The rule for extracting the subject is extracting the central region with the area size of the subject image is shown in,


$$S_{\mathrm{subject}}=\mathrm{Area}\left(\alpha ,\beta \right)=\left(\alpha *H\right)*\left(\beta *W\right)$$

where $\mathrm{Area}\left(\cdot ,\cdot \right)$ denotes the solution function to the area, and α and β are the hyperparameter ratios of length and width, respectively, set α = β .

The rule for extracting the background is extracting the four corners of the image. Afterwards, the four corners are pieced together to form a background image. The area size of the background image is shown in,


$$S_{\mathrm{background}}=\mathrm{Area}\left(\gamma ,\delta \right)=\left(\gamma *H\right)*\left(\delta *W\right)$$

where γ and δ are the hyperparameter ratios of length and width, respectively, set γ = δ .

After the sample $x_{i}$ is input into the extraction module E , two samples are obtained at the output end (the subject sample and the background sample of the original image), which is given by,


$$Sub\left(x_{i}\right)=f_{\mathrm{crop}}\left(x_{i},S_{\mathrm{subject}}\right)$$

$$Bac\left(x_{i}\right)=f_{\mathrm{crop}}\left(x_{i},S_{\mathrm{background}}\right)$$

$$x_{i\left(\mathrm{subject}\right)},x_{i\left(\mathrm{background}\right)}=Sub\left(x_{i}\right),Bac\left(x_{i}\right)$$

where $f_{\mathrm{crop}}$ is a crop function. $Sub\left(x_{i}\right)$ and $Bac\left(x_{i}\right)$ are the functions to exract the subject and background, respectively.

The three samples are further subjected to data augmentation, feature extraction, and projection mapping, and finally, three sets of feature vectors are acquired, which is given by,


$$z_{i},z_{i\left(\mathrm{subject}\right)},z_{i\left(\mathrm{background}\right)}=g\left(f\left(t\left(x_{i},x_{i\left(\mathrm{subject}\right)},x_{i\left(\mathrm{background}\right)}\right)\right)\right)$$

### 3.3 Build multi-views

The view of SimCLR (Chen et al., 2020) is constructed as follows. Firstly, randomly sample N sample images. After two random data augmentations, we obtain 2N expanded samples and two views. The two expanded samples originating from the same image are defined as mutual positive samples, and the remaining $2\left(N-1\right)$ samples are defined as the negative samples of the two positive samples.

**Figure 3:**
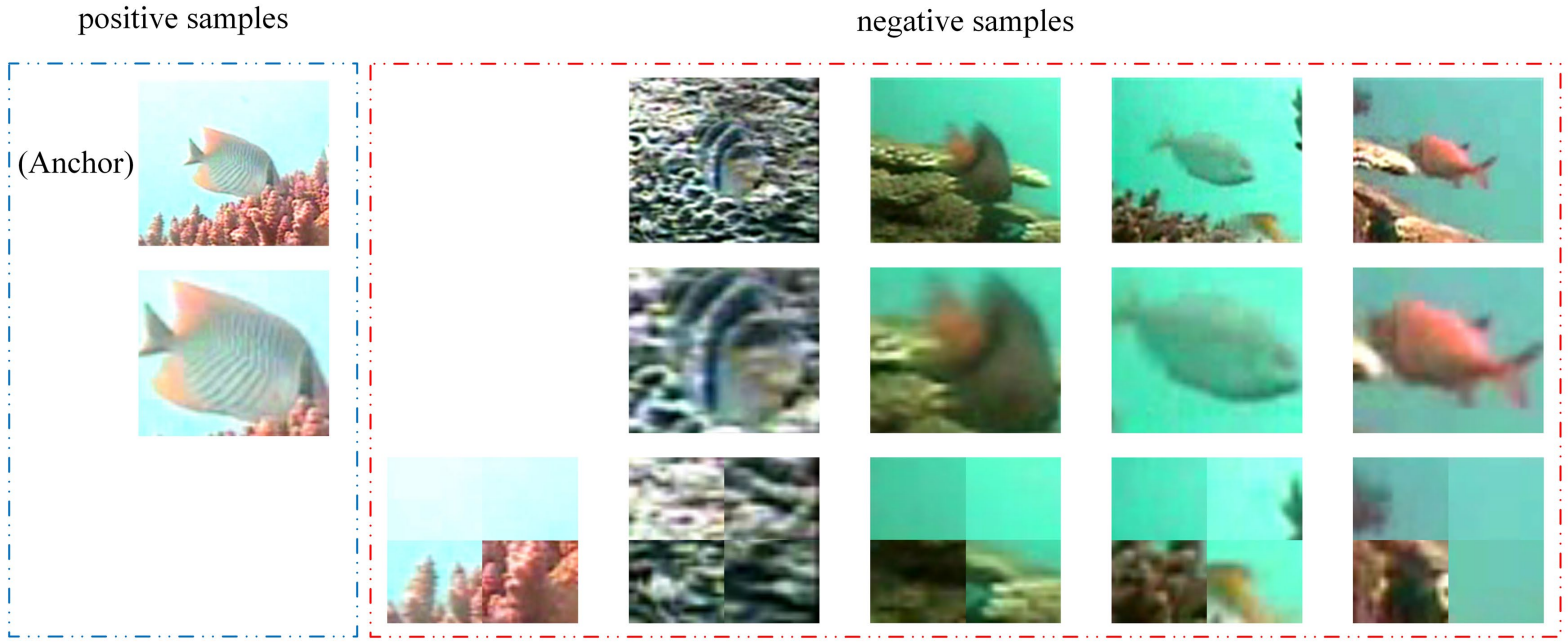
The diagram of positive and negative samples of CLIB. The first row of images are the original views, the second row of images are their respective subject views, and the third row of images are their respective background views. The upper left original image is regarded as an anchor, its subject view (the below image in blue line) is regarded as the positive sample of the anchor, and all other images are regarded as negative samples of the anchor (all images in red line).

The views of CLIB are constructed as follows. Firstly, randomly select N sample images ( N original images) and input the selected N original images into the extraction module to obtain 2N samples, i.e., N subject samples and N background samples. After randomly augmenting the 3N samples, we obtain 3N expanded samples. The expanded sample of an original image and the expanded sample of the subject sample extracted from the original image are mutual positive samples, and the rest of the 3N−2 expanded samples are defined as negative samples of the expanded sample of the original image or the subject sample. The acquired positive and negative samples are shown in Figure 3.

#### 3.3.1 Multi-view-based contrastive loss function

SimCLR (Chen et al., 2020) follows the idea of contrastive learning. After constructing two views, each sample in the two views corresponds to one similar sample (positive sample) and $2\left(N-1\right)$ dissimilar samples (negative samples). 2N feature vectors are obtained after 2N samples are encoded through an encoder and projected through a projection network, and the similarity between two feature vectors is calculated according to the cosine similarity formula, which is given by,


$$sim\left(z_{i},z_{j}\right)=\frac{z_{i}^{T}z_{j}}{\left\|z_{i}\|\|z_{j}\right\|}$$

where $z_{i}$ denotes the feature vector of sample i , $z_{j}$ denotes the feature vector of sample j , T represents the transpose of the vector, and $\left\|z_{i}\right\|$ indicates the length of the vector $z_{i}$ . For the positive samples pair $\left(i,j\right)$ , the definition of the loss function for SimCLR (Chen et al., 2020) is given by,


$$l_{i,j\left(\mathrm{SimCLR}\right)}=-\log\frac{\exp\left(sim\left(z_{i},z_{j}\right)/\tau \right)}{\sum_{k=1}^{2N}1_{\left[k\neq i\right]}\exp\left(sim\left(z_{i},z_{k}\right)/\tau \right)}$$

where $1_{\left[k\neq i\right]}\in \left\{0,1\right\}$ is an indicator function with value “1” when k≠i and value “0” when k=i , τ is a temperature parameter, and $\exp\left(\cdot \right)$ is the exponential function. The total loss is given by,


$$L_{\mathrm{SimCLR}}=\frac{1}{2N}\overset{N}{\underset{k=1}{\sum }}\left[l_{\left(2k-1,2k\right)}+l_{\left(2k,2k-1\right)}\right]$$

where $\left(2k-1,2k\right)$ and $\left(2k,2k-1\right)$ represent the pairs of positive samples.

CLIB also follows the idea of contrastive learning, but unlike SimCLR (Chen et al., 2020), the number of negative samples in CLIB increases significantly. This is because, besides the $2\left(N-1\right)$ negative samples in both the original image view and the subject view, the N samples in the background view are also defined as negative samples. After the three views are constructed, each sample in either the subject or the original image view has one similar sample (positive sample) and 3N−2 dissimilar samples (negative samples). In CLIB, 3N feature vectors are obtained after 3N samples are encoded through an encoder and projected through a projection network. For the positive sample pair $\left(i,j\right)$ and all the corresponding negative samples, the loss function of CLIB is given by,


$$l_{i,j\left(\mathrm{CLIB}\right)}=-\log\frac{\exp\left(sim\left(z_{i},z_{j}\right)/\tau \right)}{\sum_{k=1}^{3N}1_{\left[k\leq 2N,k\neq i:1;k>2N:1\right]}\exp\left(sim\left(z_{i},z_{k}\right)/\tau \right)}$$

where $1_{\left[k\leq 2N,k\neq i:1;k>2N:1\right]}\in \left\{0,1\right\}$ is the indicator function with value “1” when k≤2N and k≠i or k>2N , in all other cases, the value of the function is 0. k≤2N means that k belongs to the original images view or the subject view. If k>2N , the value of the indicator function is “1.” τ is the temperature parameter, and $\exp\left(\cdot \right)$ is the exponential function. The total loss is given by,


$$L_{\mathrm{CLIB}}=\frac{1}{2N}\overset{N}{\underset{k=1}{\sum }}\left[l_{\left(3k-1,3k-2\right)}+l_{\left(3k-2,3k-1\right)}\right]$$

where $\left(3k-1,3k-2\right)$ and $\left(3k-2,3k-1\right)$ represent the pairs of positive samples. It should be noted that the positive samples only appear in either the original view or the subject view, and all the samples in the background view are negative samples.

## 4 Experiments

In this section, the performance of CLIB is evaluated and compared with the benchmark model (SimCLR) as well as nine mainstream self-supervised visual representation learning methods through experiments in which the encoder is ResNet50 (He et al., 2016).

### 4.1 Datasets and experimental set-up

#### 4.1.1 Datasets

The experiments are performed on four datasets. The types and quantities of the four datasets are shown in Table 2. The fish images in Fish4Knowledge (Boom B. et al., 2012; Boom B. J. et al., 2012), WildFish-30, and QUTFish-89 datasets are taken in water, while the fish images in the Fish-gres dataset (Prasetyo et al., 2020) are taken on land. The WildFish-30 (Zhuang et al., 2018) dataset is composed of the images in the 30 categories with the highest number of images. The QUTFish-89 dataset is composed of the images in the 89 few-sample categories from the QUTFish dataset (Anantharajah et al., 2014).

**Table 2:** Type information of four datasets.

Dataset	Number of species	Number of images	Resized resolution
Fish4Knowledge	23	27,370	64 × 64
Fish-gres	8	3,248	224 × 224
WildFish-30	30	3,688	224 × 224
QUTFish-89	89	823	224 × 224

#### 4.1.2 Experimental set-up

The experiments in this paper are conducted under the same hardware and software environment. Specifically, the CPU used is Intel(R) Xeon(R) Platinum 8358P, while the GPU used is A40 (48GB). The size of the memory is 80GB. The Python version is 3.8, while the Pytorch version is 2.0. When training in the backbone network, all samples are put into the network for training, and the model with the lowest loss value is preserved. When training in the classifier, the dataset is divided into three subsets with a ratio of 1:1:8. One subset with 10% samples is used as the training set, another subset with 10% samples is used as the validation set, and the remaining subset with 80% samples is used as the test set. The model with the highest accuracy in the classifier training process on the validation set is reserved, and the final accuracy is obtained by testing the test set with the reserved model. The rest of the experimental setup is shown in Table 3.

**Table 3:** Experimental set-up.

Environment and parameters	Set-up
Optimizer	SGD
Initial learning rate	0.01
Final learning rate	0.0001
Temperature	0.07
Training epochs of the backbone network	500
Training epochs of the classifier	100

### 4.2 Results of comparative experiments

To verify the effectiveness and superiority of the CLIB method, nine popular self-supervised methods of visual representation learning are selected for comparison, including SimCLR (a simple framework for contrastive learning of visual representations; Chen et al., 2020), MOCO (momentum contrast for unsupervised visual representation learning; He et al., 2020), SimSiam (exploring simple siamese representation learning; Chen and He, 2021), BYOL (bootstrap your own latent; Grill et al., 2020), TiCo (transformation invariance and covariance contrast for self-supervised visual representation learning; Zhu et al., 2022), NNCLR (nearest-neighbor contrastive learning of visual representations; Dwibedi et al., 2021), Dcl (decoupled contrastive learning; Yeh et al., 2022), Matrix-SSL (Matrix Information Theory for Self-Supervised Learning; Zhang et al., 2023), and Mixed Barlow Twins (Guarding Barlow Twins Against Overfitting with Mixed Samples; Bandara et al., 2023). To test the proposed CLIB method more objectively, to define four metrics, Accuracy, Precision, Recall, and F1 value, as the metrics to evaluate the classification performance of the methods.

#### 4.2.1 Comparative experiments on underwater images with different resolutions

**Figure 4:**
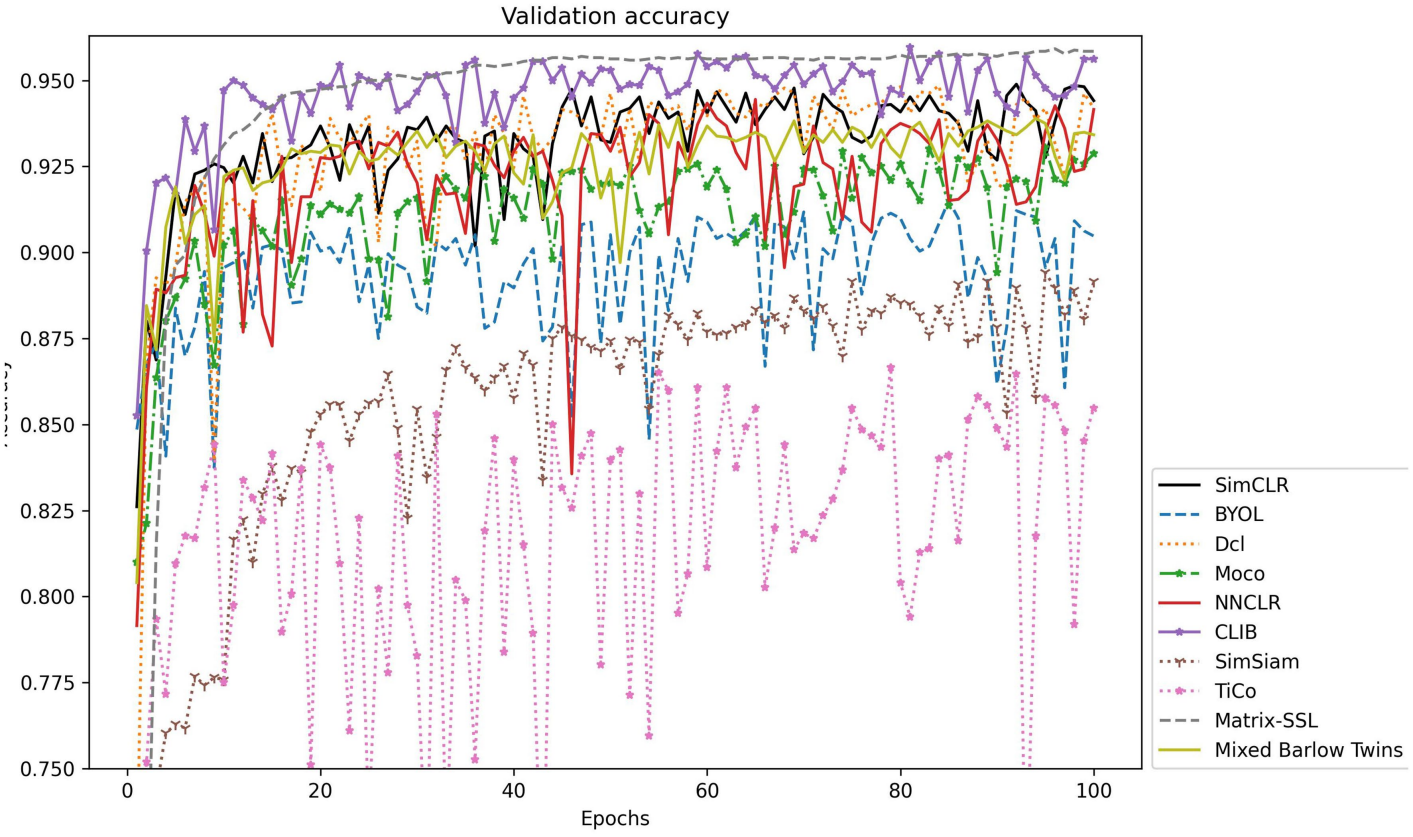
Accuracy validation on the Fish4Knowledge dataset.

**Figure 5:**
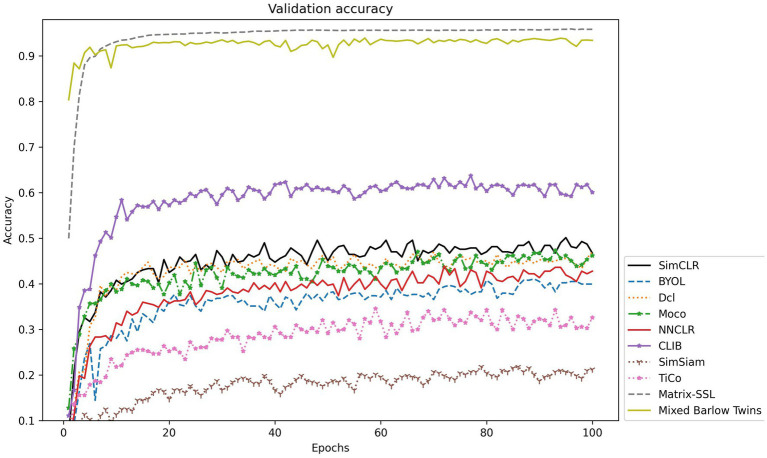
Accuracy validation on the WildFish-30 dataset.

To further explore the actual effect of CLIB, we conduct experiments on Fish4Knowledge and WildFish-30 datasets with very different resolutions, and the results are shown in Table 4. Meanwhile, the validation accuracy is shown in Figures 4, 5.

**Table 4:** Experimental results on the Fish4Knowledge and WildFish-30 datasets.

Method	Fish4Knowledge				WildFish-30			
	Acc (%)	Pre (%)	Rec (%)	F1 (%)	Acc (%)	Pre (%)	Rec (%)	F1 (%)
SimCLR (Chen et al., 2020)	94.58	86.83	69.19	74.10	46.56	49.88	46.17	46.82
Moco (He et al., 2020)	93.12	78.78	66.02	69.06	44.55	46.50	44.48	44.33
SimSiam (Chen and He, 2021)	88.28	41.32	34.57	35.33	21.99	24.35	21.94	21.10
BYOL (Grill et al., 2020)	91.01	60.97	46.82	48.54	39.55	41.65	39.32	39.52
TiCo (Zhu et al., 2022)	86.36	58.63	48.30	49.20	32.48	34.42	32.21	31.16
NNCLR (Dwibedi et al., 2021)	94.07	69.17	66.47	65.93	41.60	44.75	41.45	41.53
Dcl (Yeh et al., 2022)	94.83	85.43	71.19	74.60	45.02	46.43	44.78	44.60
Matrix-SSL (Zhang et al., 2023)	95.93	70.80	56.56	59.70	40.96	42.41	40.78	41.02
Mixed Barlow Twins (Bandara et al., 2023)	93.21	53.25	47.28	47.86	33.48	36.24	33.18	32.51
CLIB	96.01	91.48	75.94	80.16	60.94	62.98	60.99	61.44

The experimental results show that the CLIB method performs excellently on the lower-resolution Fish4Knowledge dataset and the higher-resolution WildFish-30 dataset. It is worth noting that the effect of CLIB on the higher-resolution WildFish-30 dataset is more prominent. This is because the original image resolution is high, CLIB extracts more pixel points of the subject and background image parts, which provides the model with richer information about the negative samples compared to other methods and makes the model pay attention to the subject part of the image and ignore the background part. In conclusion, experimental results and analysis on the Fish4Knowledge and WildFish-30 datasets show that CLIB performs excellently in underwater fish image classification of different resolutions with background noise.

#### 4.2.2 Comparative experiments in complex backgrounds

**Figure 6:**
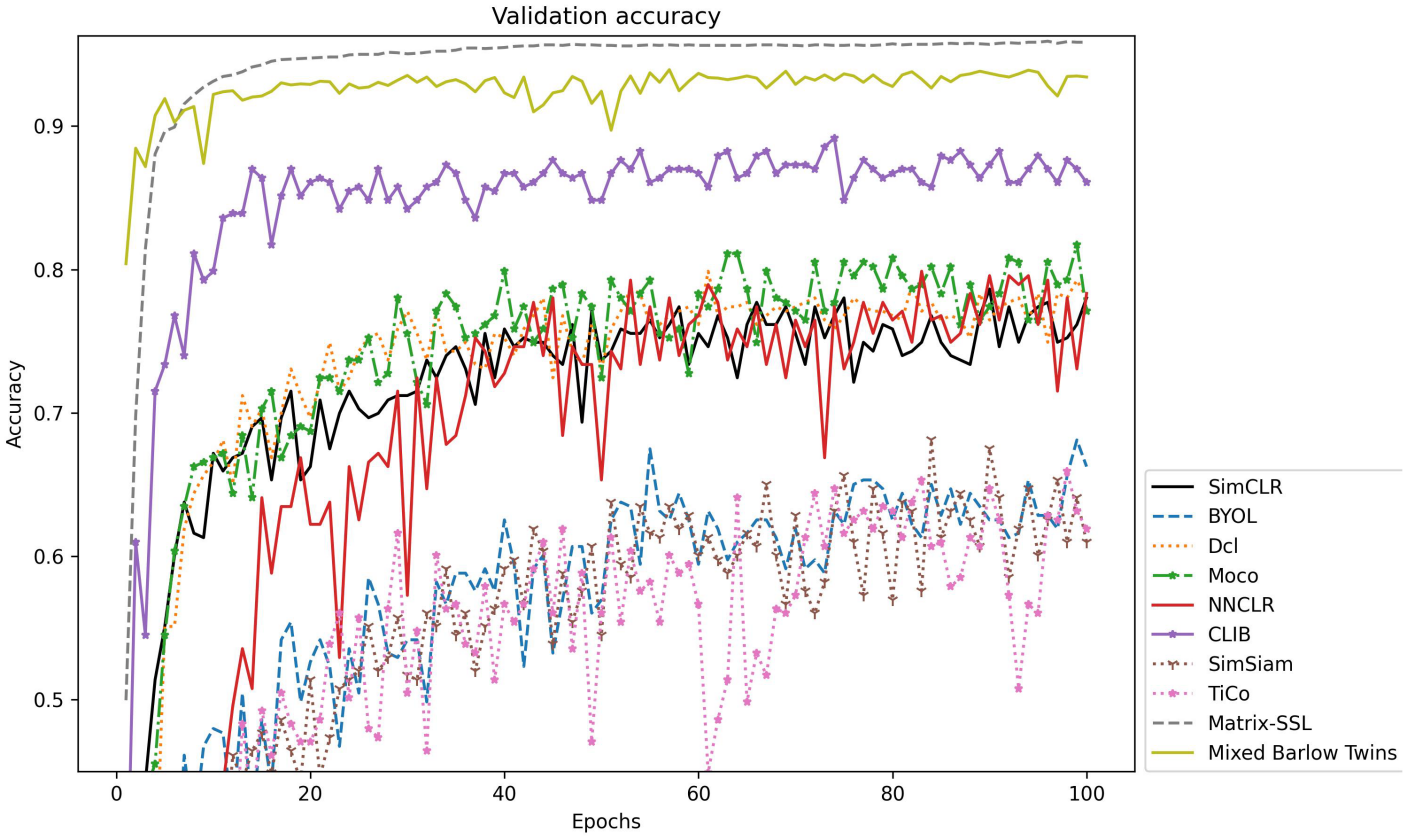
Accuracy validation on the Fish-gres dataset.

To reflect the main idea that CLIB focuses more attention on the subject of the image and ignores the background, we purposely conduct experiments on the dataset of Fish-gres, in which the fish images are taken from land, and the backgrounds of images of fishes belonging to the same species are different greatly. The experimental results on the Fish-gres datasets are shown in Table 5, and the validation accuracy is shown in Figure 6.

**Table 5:** Experimental results on the Fish-gres dataset.

Method	Fish-gres			
	Acc (%)	Pre (%)	Rec (%)	F1 (%)
SimCLR (Chen et al., 2020)	78.87	80.22	77.45	78.22
Moco (He et al., 2020)	78.98	80.35	77.88	78.84
SimSiam (Chen and He, 2021)	66.65	66.61	64.73	65.38
BYOL (Grill et al., 2020)	68.03	70.14	64.65	65.98
TiCo (Zhu et al., 2022)	67.96	69.01	66.89	66.44
NNCLR (Dwibedi et al., 2021)	80.17	81.43	80.10	80.32
Dcl (Yeh et al., 2022)	77.75	80.82	76.28	77.96
Matrix-SSL (Zhang et al., 2023)	74.10	74.11	71.04	71.94
Mixed Barlow Twins (Bandara et al., 2023)	59.16	62.16	55.30	55.90
CLIB	87.59	88.38	86.35	87.17

For datasets like Fish-gres with large background differences between similar classes, it should be more difficult for the ordinary contrastive learning methods to achieve feature learning by zooming in the feature mapping of positive sample pairs and zooming out the feature mapping of negative samples. In this paper, we propose the CLIB method. The main idea of CLIB is to pay more attention to the subject of the image and ignore the background of the image. Theoretically, CLIB should perform much better on the Fish-gres dataset. The experimental results show that CLIB achieves accuracy of 87.59%, precision of 88.38%, recall of 86.35%, and F1 value of 87.17% on the dataset of Fish-gres, which outperforms SimCLR (8.72%), Moco (8.61%), SimSiam (20.94%), BYOL (19.56%), TiCo (19.63%), NNCLR (7.42%), Dcl (9.84%), Matrix-SSL (13.49%), and Mixed Barlow Twins (28.43%) in terms of accuracy. Furthermore, CLIB outperforms the nine methods in terms of precision, recall, and F1 value, which verifies the validity of the CLIB’s idea of ignoring the background and focusing on the subject.

#### 4.2.3 Comparative experiments with few-sample datasets

**Figure 7:**
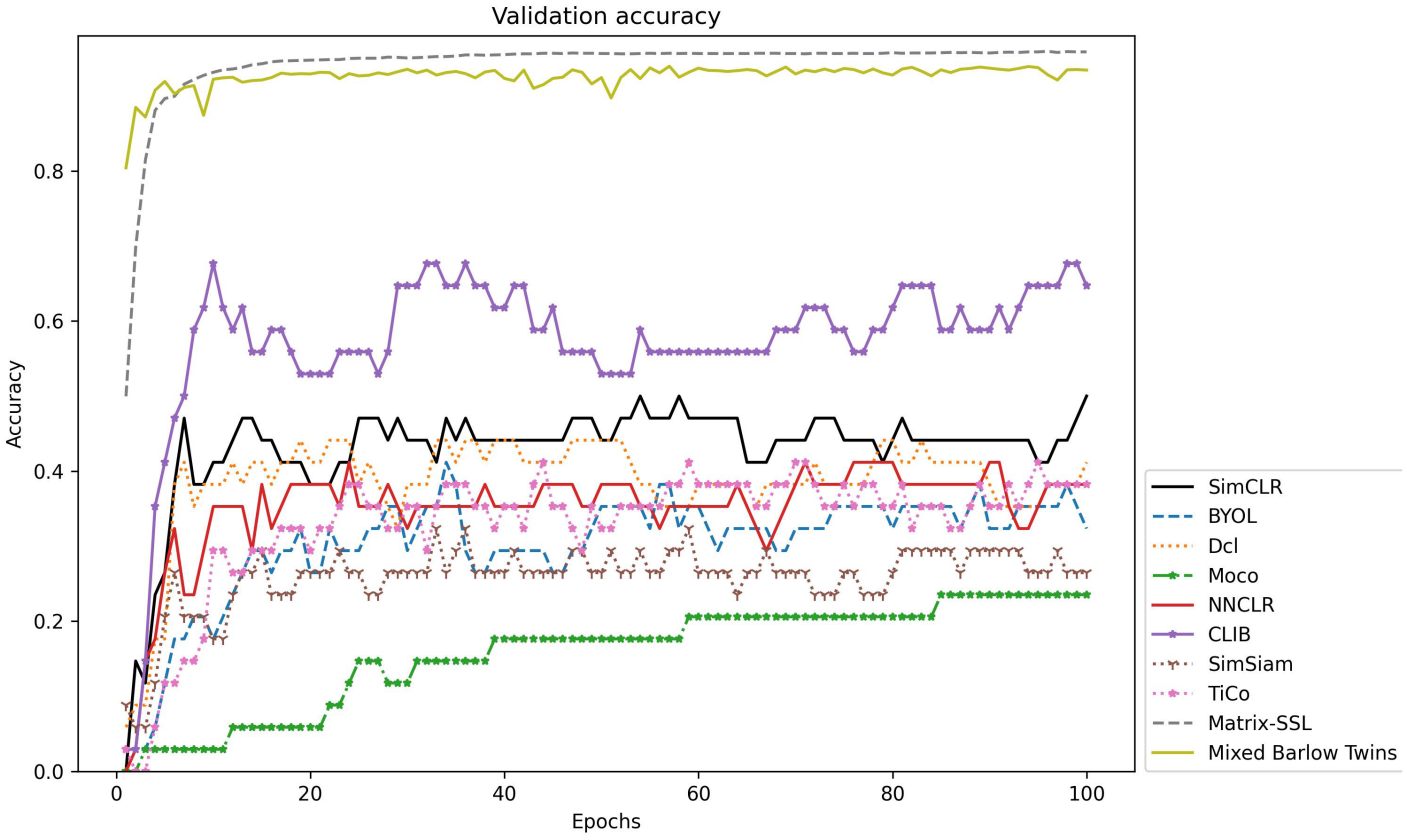
Accuracy validation on the QUTFish-89 dataset.

When taking underwater fish images, it is often difficult to capture enough fish images due to the sparse number of fish in some species, resulting in some categories of the dataset presenting a low sample size. To better adapt to this situation, we further evaluate CLIB’s ability to learn with few samples and its generalization by constructing a few-sample dataset. Eighty-nine categories with few samples are extracted from the QUTFish dataset to form the QUTFish-89 dataset, most of which have less than 10 images in the category. Not only that, only 34 images were used to fine-tune the model for the classifier, and far fewer than the categorized categories 89. The experimental results on the QUTFish-89 dataset are shown in Table 6, and the validation accuracy is shown in Figure 7.

**Table 6:** Experimental results on the QUTFish-89 dataset.

Method	QUTFish-89			
	Acc (%)	Pre (%)	Rec (%)	F1 (%)
SimCLR (Chen et al., 2020)	10.71	6.98	9.16	6.39
Moco (He et al., 2020)	4.36	3.60	3.90	2.68
SimSiam (Chen and He, 2021)	5.29	4.21	4.62	3.33
BYOL (Grill et al., 2020)	6.87	4.79	5.74	4.04
TiCo (Zhu et al., 2022)	6.21	4.64	5.49	3.54
NNCLR (Dwibedi et al., 2021)	7.53	6.12	6.73	4.63
Dcl (Yeh et al., 2022)	8.99	5.94	7.88	5.22
Matrix-SSL (Zhang et al., 2023)	11.11	6.69	9.65	6.53
Mixed Barlow Twins (Bandara et al., 2023)	6.21	6.88	5.60	3.51
CLIB	16.13	13.17	13.89	10.31

Under such demanding conditions, the CLIB method still obtains good results compared to the other nine methods, with significant advantages in all four metrics. This is because in the training phase of the backbone network, benefiting from the idea of focusing on the subject of the image and ignoring the background of the image, CLIB is less affected by the background in the process of learning, and the model can more accurately capture the differences between different categories of images and the sameness between the same categories. As a result, CLIB can also distinguish different categories of fish images well when faced with this sparse number of samples. At the same time, the other nine methods make it more difficult for the model to learn the homogeneity between the same categories when facing image data with sparse samples because of the scarcity of data in the same category. In addition, the backgrounds of fish images between different categories are highly similar, while fish images of the same category can have large differences, and the sparse data make it more difficult for these methods to learn the differences between images of different categories and the homogeneity between the same categories.

### 4.3 Results of ablation experiments

To further verify the superiority of CLIB, three groups of ablation experiments are designed:

#### 4.3.1 Baseline

The experiments of the first group are conducted with the baseline model SimCLR, which consists of two views of the two original images derived from the same image with two different data enhancements.

#### 4.3.2 CLIB-ablation

The experiments of the second group, the background view is added in the SimCLR model as expanded negative samples of two enhanced image views, and forms the third view.

#### 4.3.3 CLIB

The experiments of the third group are conducted with the proposed CLIB method, with three views in total: original view, body view, and background view.

The results of the ablation experiments are shown in Tables 7, 8. The experiment results of the second group are either better or worse than those of the first group. This is because in the second set of methods, on the one hand, the mappings in the feature space between one of the enhanced original views and the background view is zoomed out in the process of training, which results in the model ignoring the background. On the other hand, the mappings in the feature space between one enhanced original image and another enhanced original image is zoomed in during the training process. However, due to the two images in the two original views being with background, the mapping between the two backgrounds in the two original views is also zoomed in, which results in the model failing to ignore the background. Therefore, it can be concluded that simply adding background views to SimCLR to expand the negative samples does not improve the performance of the model.

**Table 7:** Experimental results on Fish4Knowledge and Fish-gres datasets.

Method	Fish4Knowledge				Fish-gres			
	Acc (%)	Pre (%)	Rec (%)	F1 (%)	Acc (%)	Pre (%)	Rec (%)	F1 (%)
Baseline	94.58	86.83	69.19	74.10	78.87	80.22	77.45	78.22
CLIB-ablation	94.73	81.22	70.57	73.72	79.40	80.03	78.68	79.07
CLIB	96.01	91.48	75.94	80.16	87.59	88.38	86.35	87.17

**Table 8:** Experimental results on WildFish-30 and QUTFish-89 datasets.

Method	WildFish-30				QUTFish-89			
	Acc (%)	Pre (%)	Rec (%)	F1 (%)	Acc (%)	Pre (%)	Rec (%)	F1 (%)
Baseline	46.56	49.88	46.17	46.82	10.71	6.98	9.16	6.39
CLIB-ablation	48.44	49.50	48.08	48.09	12.43	8.93	10.70	7.56
CLIB	60.94	62.98	60.99	61.44	16.13	13.17	13.89	10.31

The ablation experiments of the third group are conducted using the CLIB method proposed in this paper. Consequently, compared to the experiments in the first or second group, the performance of the CLIB method is significantly improved, which is verified by the experimental results in Tables 7, 8. This is because the CLIB method does not suffer from the contradiction in the second set of experiments. Thus, the ablation experiments verify the validity of the idea of the CLIB by focusing on the subject and ignoring the background.

### 4.4 Visualization

**Figure 8:**
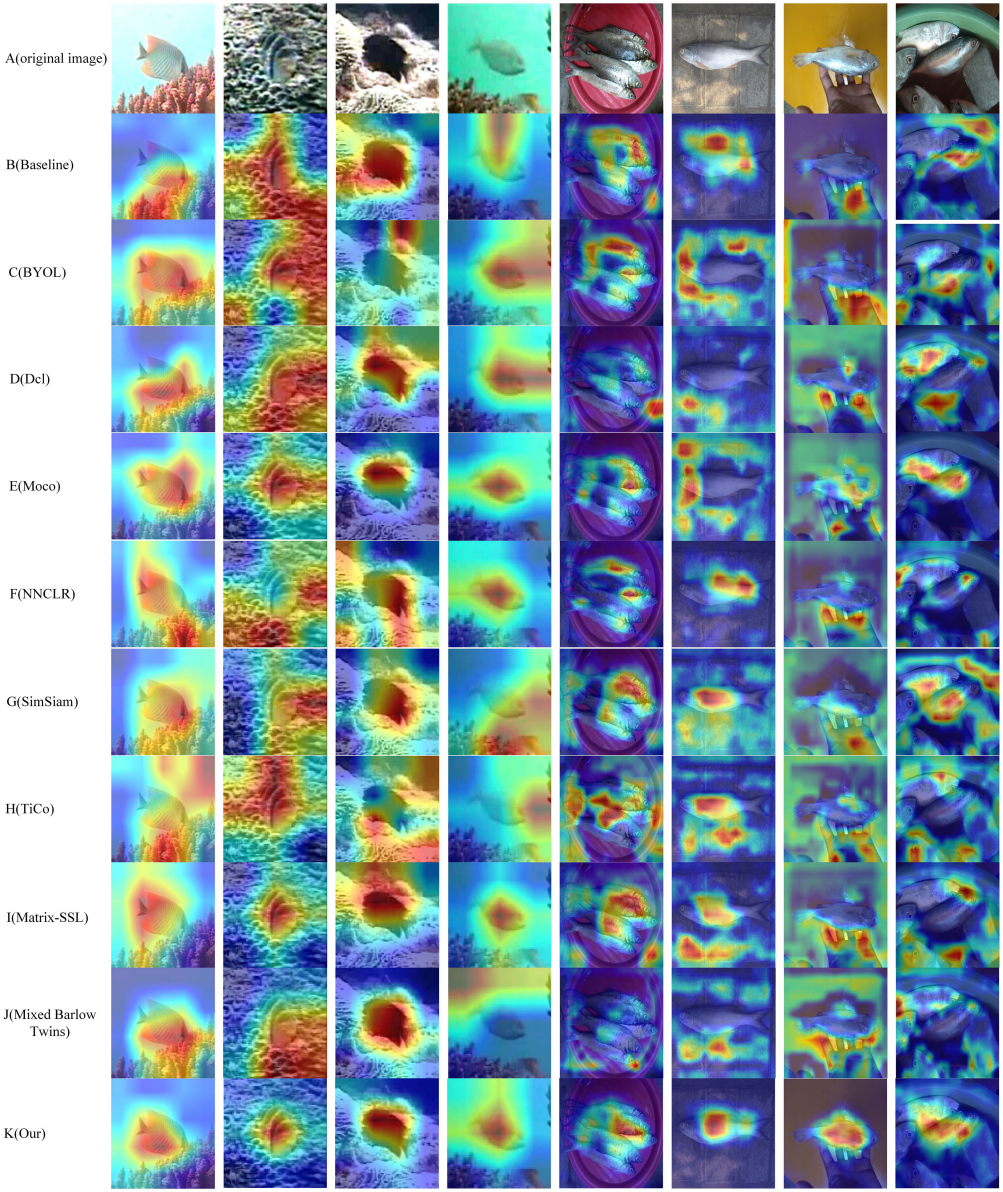
Network attention visualization. The redder the area, the more critical it is for decision or classification.

To visualize the idea of CLIB of focusing on the subject and ignoring the background, experiments on visualizing network attention using class activation maps (Grad-Cam; Selvaraju et al., 2017) are conducted. The results are shown in Figure 8. The redder the area, the more critical it is for decision or classification, and the SimCLR model is used as the baseline in the visualization experiment. Most general methods tend to regard the background in the image as part of the fish for classification. Figure 8 shows the subject area is concerned with both the benchmark model and CLIB. However, with the benchmark model, the recognition results are easily affected by backgrounds. For example, in the first row, although the benchmark model focuses on the fish body of the image, it also focuses on the grass under the fish. In the second row, the subject of the image is similar to the background, which makes it challenging to locate the fish even with the human eye. Since the baseline model cannot accurately differentiate between the subject and the background, the baseline model incorrectly regards the grass that is highly similar to the fish as the subject of the image. However, this is not the case for CLIB. The CLIB model accurately draws the outline of the fish. Similarly, from the visualization results on the Fish-gres dataset in the seventh row, the baseline model focuses mainly on the wrist and ignores the fish in the hand, while CLIB can accurately focus on the fish in the image. In summary, the superiority of the CLIB is further verified by visualizing experiments on network attention using class activation graphs.

### 4.5 Analysis of parameter sensitivity

**Figure 9:**
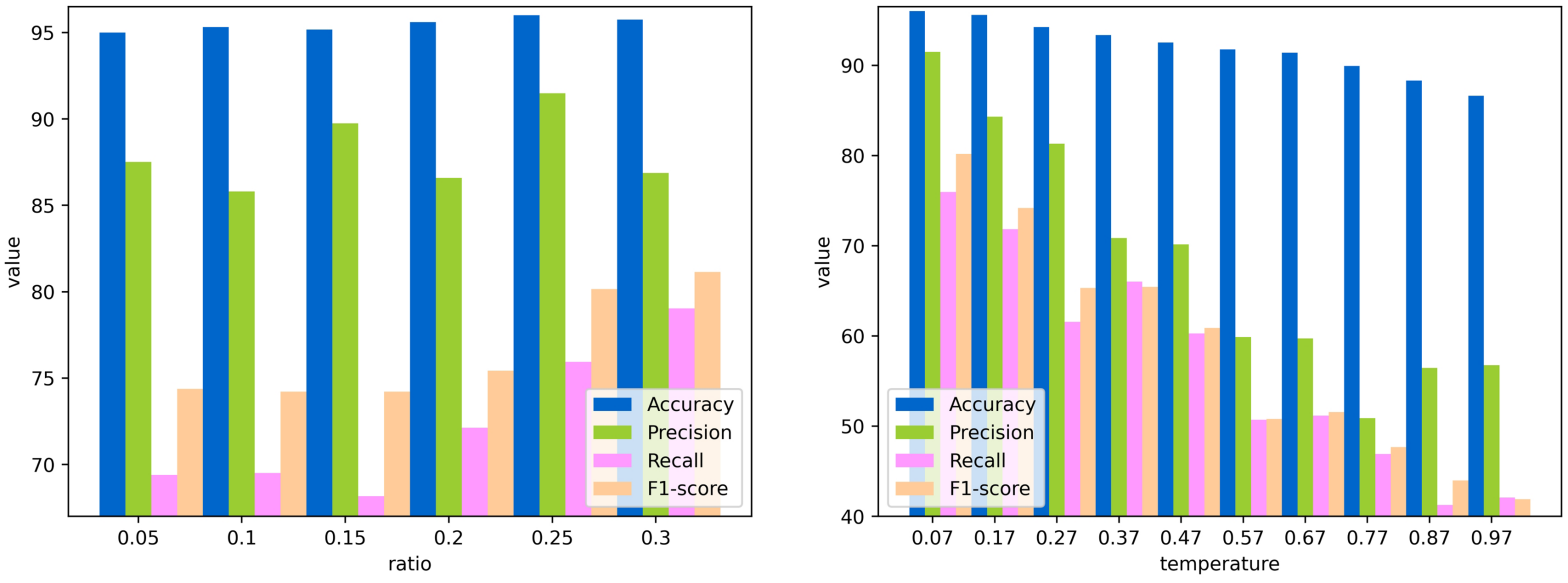
Performance of CLIB under different ratios and temperatures. **(A)** Performance under different ratios. **(B)** Performance under different temperatures.

In this subsection, the performance of CLIB under different settings of parameters is shown in Figure 9. It is worth stating that the experiments are conducted on the dataset of Fish4Knowledge with resnet50 as the backbone network, and experiments can be performed under the same settings of parameters on other datasets and backbone networks as well.

The first parameter is the ratio of α to γ of half of the side length of the background image to the side length of the original image in the extraction module. Figure 9 shows the performance of CLIB changes with the ratio, and the most effective ratio is 0.25. This is because when the ratio is 0.25, the size of the mosaic of backgrounds at the four corners of the original image is equal to that of the original image. Thus, more pixel points can be used in contrastive learning, and the result is better.

**Figure 10:**
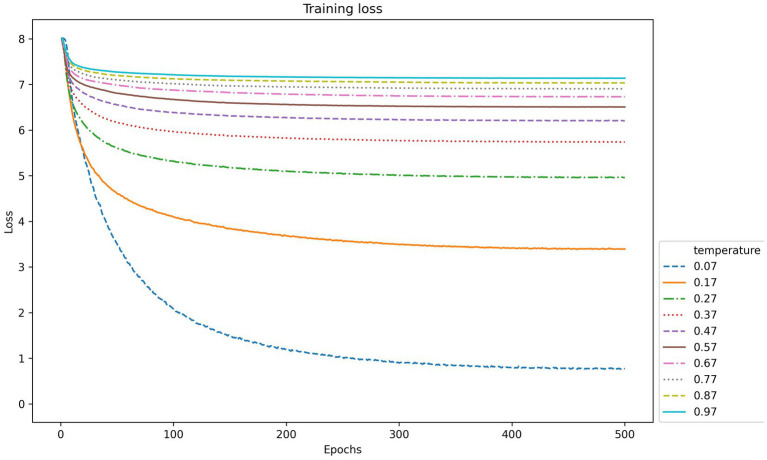
The value of loss function in backbone network training of CLIB under different temperatures and epochs.

The second parameter is the temperature in contrastive learning, which is used to control the model’s ability to differentiate between positive and negative samples. The performance index values of CLIB in the training phase of the backbone network under different temperature parameters are shown in Figures 9, 10. It is seen that the higher the temperature parameter is, the weaker ability to differentiate the positive and negative samples of the model is, and the higher loss value in the training process of the model is. However, if the temperature is set very small, the model is difficult to converge or has poor generalization ability.The most effective temperature coefficient of CLIB is 0.07.

## 5 Conclusion

In this paper, to solve the problem of background noise having a tremendous negative impact on underwater fish image classification, we propose the contrastive learning method of ignoring background for underwater fish image classification called CLIB. CLIB redefines views and loss function in contrastive learning based on the characteristics of underwater fish images. We demonstrate the effectiveness of CLIB in underwater fish image classification, especially when facing different resolutions, complex backgrounds, and few-sample. When the subject is located in the center of the image, the proposed CLIB method achieves the best classification effect. However, if the fish is located in a corner of the image, the CLIB treats the fish as the background, and the classification effect is decreased, which is a problem we need to solve in our subsequent work.

## Data availability statement

The original contributions presented in the study are included in the article/supplementary material, further inquiries can be directed to the corresponding authors.

## Author contributions

QY: Conceptualization, Methodology, Writing – original draft. XD: Funding acquisition, Methodology, Writing – original draft. CL: Software, Writing – review & editing. XT: Conceptualization, Writing – review & editing.
